# Editorial: Advancing soft, tactile, and haptic technologies: recent developments for healthcare applications

**DOI:** 10.3389/frobt.2025.1658613

**Published:** 2025-08-08

**Authors:** Antonia Tzemanaki, Benjamin Ward-Cherrier, Matteo Cianchetti, Jelizaveta Konstantinova

**Affiliations:** ^1^ School of Engineering Mathematics and Technology, Bristol Robotics Laboratory, University of Bristol, Bristol, United Kingdom; ^2^ The BioRobotics Institute, Scuola Superiore Sant’Anna, Pisa, Italy; ^3^ Ocado Technology, Welwyn Garden City, United Kingdom

**Keywords:** soft robotics, tactile sensing, haptics, healthcare technologies, medical robotics and systems

## Introduction

Recent advancements in soft robotics, tactile sensing, and haptic technologies are opening new frontiers in healthcare, particularly in enhancing diagnostic precision, clinical training, patient monitoring, and human-robot interaction (Raman and Laschi, 2024; Othman et al., 2022; Zhu et al., 2022).

Using soft and smart materials, robotic mechanisms and sensors become compliant and deformable, mimicking the flexibility and adaptability of natural organisms. These characteristics can advance tactile sensors and haptic systems to enable robots to perceive and interact with their environment through touch in a safer and seamless manner, while also elevating human-robot interaction to a near-natural and intuitive experience (Yin et al., 2021; Zhou et al., 2024).

There is potential to revolutionize healthcare via offering innovative solutions that improve patient outcomes, enhance healthcare professionals’ skills, and provide personalized and adaptive care. Recent developments such as variable stiffness mechanisms (Fras et al., 2021) and compliant soft grippers for manipulation or safer and more flexible surgical procedures (Pagliarani et al., 2023) as well as sensor tuning techniques (Jenkinson et al., 2023) aim to enhance the capabilities of these technologies.

This Research Topic explores the challenges and opportunities of these areas, highlighting state-of-the-art methodologies and innovations in soft tactile sensors, wearable haptic devices, multimodal learning frameworks, and intelligent systems for healthcare. The contributions collectively demonstrate how soft tactile and haptic technologies are evolving to meet the demands of modern medicine through adaptive, accessible, and context-aware solutions.

## Contributed articles

The study by Samain-Aupic et al. offers compelling insights for the field of soft, tactile, and haptic technologies in healthcare by revealing how tactile sensitivity evolves across the human lifespan and body. Showing that glabrous skin (e.g., of the fingertip) deteriorates with age while hairy skin (e.g., cheek and forearm) retains sensitivity, this paper highlights the importance of tailoring haptic interfaces to different skin types and age groups. These findings can directly inform the design of next-generation wearable sensors, prosthetics, and rehabilitation tools, ensuring they remain effective and perceptible for older users. As healthcare increasingly embraces tactile feedback systems, understanding the nuanced aging of touch becomes essential for creating inclusive, responsive, and human-centred technologies.

Palpation is an essential skill also for clinicians and other medical professionals that aids diagnostics and physical examination of patients. High-fidelity human manikins are costly and not broadly available, especially for developing countries. Brown and Bello present the development of four particle-jamming–based tactile displays for simulating organic soft-tissue hardness of different properties. The authors describe electromechanical constriction and compression devices, a magnet-based design, and a vacuum approach. Notably, apart from the quantitative performance evaluation, they also consider non-functional characteristics such as size and cost, informing further development of the proposed innovation. The described tactile display systems provide insights for the development of low-cost palpation simulators, making realistic clinical skills practice accessible even in resource-limited settings.

A key topic in tactile sensing systems is their integration with other artificial sensory modalities such as vision. Li and Thuruthel introduce a self-supervised multimodal learning framework that fuses vision and tactile data to improve robotic interaction prediction in dynamic tasks. The system collects synchronized vision, tactile and action data and reveals the asymmetric and complementary roles of vision and touch, with tactile input proving especially valuable under visual occlusion. This offers important insights into how vision and tactile sensors could be combined for dexterous manipulation tasks, particularly in healthcare.

The paper by Gandhi et al. presents an optical soft tactile sensing system designed for head motion tracking in radiotherapy settings, offering a non-metallic, MRI-compatible alternative to conventional vision-based systems. The authors develop and evaluate a Motion Capture Pillow that uses a fibrescope and optical flow algorithms (Lukas-Kanade) to detect head movements with high fidelity. This work enables contact-based tracking in electromagnetically sensitive healthcare environments.

Kurogi et al. introduce a novel approach for Haptic Augmented Reality that augments the perceived stickiness of a physical object during adhesion-separation contact mode. The study hypothesises that the intensity of perceived stickiness depends on the duration required for the attached object to completely detach from the skin, rather than on the force exerted. A dielectric elastomer actuator based thin-film, soft wearable tactile display has been developed to introduce vibration and evoke the sensation of complete detachment of the object from the skin, thereby altering the perceived stickiness. The system has been assessed through two user experiments: the first aimed at investigating the modulation of perceived stickiness and the second to study various frequencies and presenting timings. They demonstrated that stickiness was perceived across all feedback conditions, and that realism and harmony of the experience were influenced by the frequency and presentation timing of the additional tactile feedback.

L’Orsa et al. presents a leap forward in emergency medicine by transforming how needle decompression is performed with the aim of assisting operators in detecting needle entry into the pleural space while treating tension pneumothorax. By embedding high-pass filtering and diffuse reflectance sensing into instrumented needles, this paper presents a powerful synergy with data-driven puncture detection algorithms. The system improves detection precision by up to 5.1 times compared with traditional force-only methods, while it is a lightweight, portable solution ideal for high-stakes, pre-hospital scenarios. The evaluation of multimodal sensing and ensemble techniques showcases a compelling path toward smarter, safer, and more autonomous needle-based interventions.

## Conclusion

This Research Topic underscores the transformative role of tactile and haptic technologies in healthcare, with a strong emphasis on personalization, accessibility, and multimodal integration. Central to these advancements is the adaptation of tactile systems to human variability, guiding the design of inclusive and responsive haptic interfaces. Affordable innovations are expanding access to high-fidelity clinical training and emergency care. The integration of tactile sensing with other modalities, particularly vision, enhances robotic dexterity and interaction prediction, especially in visually occluded environments. Progress in soft robotics and wearable haptics also contribute to patient-centred and patient-friendly care, offering non-invasive, MRI-compatible solutions for real-time monitoring. Collectively, these studies point to a shift toward adaptable, multimodal, and context-aware technologies poised to redefine clinical practice and patient experience.
